# Fluvoxamine ameliorates oxidative stress and inflammation induced by bile-duct ligation in male rats

**DOI:** 10.1016/j.heliyon.2022.e12344

**Published:** 2022-12-15

**Authors:** Zahra Barmoudeh, Hossein Sadeghi, Izadpanah Gheitasi, Bahman Khalvati, Navid Omidifar, Mahdokht Azizi, Zahra Moslemi, Jafar Nikbakht, Amir Hossein Doustimotlagh

**Affiliations:** a Student Research Committee, Yasuj University of Medical Sciences, Yasuj, Iran; b Medicinal Plants Research Center, Yasuj University of Medical Sciences, Yasuj, Iran; c Biotechnology Research Center, Department of Pathology, School of Medicine, Shiraz University of Medical Sciences, Shiraz, Iran

**Keywords:** Oxidative stress, Cholestasis, Fluvoxamine, Antioxidant, Inflammation

## Abstract

**Introduction:**

Cholestasis is a disorder that the bile ducts were narrowed and bile acids are not released simply. Bile acids-induced liver damage is exacerbated by inflammation and oxidative stress. The goal of the current study was to investigate the protective impacts of fluvoxamine (Flu) on oxidant-antioxidant balance and inflammatory cytokines in the bile duct ligated (BDL) rats.

**Methods:**

Thirty-two male rats were arbitrarily allocated in 4 groups; sham-control (SC), SC+ 150 mg/kg Flu (SCF), bile duct ligation (BDL), and BDL+ 150 mg/kg Flu (BDLF). The rats received distilled water and Flu orally for one week. Biochemical analysis, hematoxylin and eosin staining, as well as oxidant/antioxidant status were evaluated. Also, the mRNA expression of TGF-β1, IL-1, TNF-α, and α-SMA were determined.

**Results:**

The findings indicated serum values of ALT, total bilirubin, and ALP slightly declined in the BDL + Flu group in contrast to BDL rats. The plasma protein carbonyl and inflammatory markers were markedly increased in the BDL group in contrast with SC group (P ≤ 0.05). Treatment with Flu in BDL rats markedly reduced the values of hepatic nitric oxide metabolite and malondialdehyde, plasma protein carbonyl, as well as TNF-α mRNA level (P ≤ 0.05). Histological parameters were improved in the BDL + Flu group in comparison to BDL merely rats.

**Conclusion:**

It seems that Flu declined oxidative stress probably by inhibiting lipid peroxidation, protein oxidation, and nitric oxide formation. Also, it reduced inflammation by decreasing TNF-α mRNA expression.

## 1 Introduction

Dysfunction of the liver tissue results in acute or chronic liver diseases (CLD) ranging from fibrosis, hepatitis, cirrhosis, and hepatocellular carcinoma [1]. CLD is increasing globally, and it is a main cause of morbidity and mortality in the world [2]. Bile duct ligation (BDL) is a model of cirrhosis in rats, wherever complete biliary ligation lead to cholestatic damage [3]. The hepatic cholestasis caused by dysfunction of the liver-biliary system. This disorder results from the gathering of bile fatty acids and other toxins in the liver and plasma, if left untreated, lead to liver failure, and cirrhosis [4]. Cholestasis is well-defined as an increased levels of gamma-glutamyl transpeptidase, alkaline phosphatase, total bile acid or conjugated bilirubin in the blood [5].

The mechanism of cholestatic disease is not well understood [6]. Previous studies have shown that reactive oxygen species (ROSs) play an essential role in cholestatic damage [7]. Elevated oxidative stress and lipid peroxidation were occurred in the animal models of cholestatic disease. The base of oxidative stress in the liver of cholestatic patients is over formation of ROS. Increased levels of ROSs such as hydroxyl radical, superoxide anion, and H_2_O_2_, were reported in the blood of CLD and cholestatic patients, as well as animal models of liver diseases [8, 9]. ROSs could effect on the function of liver, kidney and other extra hepatic tissues [10]. Despite the fact oxidative stress products interact to macromolecules such as proteins, lipids, or DNA, proteins are more at risk, as a result of their role as enzymes in the cells. Protein oxidation directly impact on cell signaling, and enzymes metabolism [11]. Inflammation-induced cholestasis is common in the patients with inflammatory processes or extrahepatic infections [12]. Inflammation and Kupffer cell activation play a major role in the fibrogenesis. The Kupffer cells produce proinflammatory mediators such as IL-6, TNF-α, and IL-1, then, these cytokines effect on hepatic satellite cells [13]. Also, cytokines are involved in triggering and activating of hematopoietic stem cells, which produce collagen.

Previous study has shown that N-acetylcysteine and imipramine possesses antidepressant-like effect by drop of biochemical markers and oxidative stress [14]. Antidepressant drugs such as fluvoxamine (Flu) demonstrated potent antioxidant and anti-inflammatory effects [15, 16]. Flu is belonging to selective serotonin reuptake inhibitor (SSRI), it is commonly consumed in depression and different anxiety complaints. Flu reduced the values of the oxidant markers such as myeloperoxidase and malondialdehyde, and inhibits the CYP 1A enzyme in the stomach tissues of the animals [16]. Flu has high affinity to the 5-hydroxytryptamine receptor, and low affinity to dopamine and noradrenaline receptors, so, it has diverse side effects in contrast with monoamine oxidase inhibitors and older tricyclic antidepressants [17].

Since the anti-inflammatory and antioxidant activity of Flu in cholestasis animal models has not been investigated, the current study designed to evaluate the protective impacts of Flu on liver function, oxidant-antioxidant balance, and inflammatory cytokines in BDL cholestatic rats.

## 2 Material and methods

### 2.1 Chemicals

5,5′-dithiols-(2-nitrobenzoic acid) (DTNB), 2,4,6-tris (2-pyridyl) -s-triazine (TPTZ), and thiobarbituric acid (TBA) were obtained from Sigma Chemical Co (St Louis, MO, USA). Hydrochloric acid (HCL), trichloroacetic acid (TCA), formaldehyde, ethylenediaminetetraacetic acid (EDTA) and 2, 4-dinitrophenylhydrazine (DNPH) were gained from Merck (Germany). Fluvoxamine was purchased from Abidi Pharmaceutical Co (Tehran, Iran).

### 2.2 Experimental procedure

Thirty-two male Wistar rats (200–250 gr) were provided from Shahrekord University of Medical Sciences and adapted for 1 week in the animal laboratory. The animals were handled according to the “Principles of Laboratory Animal Care” (NIH Publication No. 86-23) and kept in the 12-hour light/dark cycle condition with free access to food and tape water (Ethical code; IR.YUMS.REC.1398.124). The animals were arbitrarily separated in 4 groups; group I (sham control, SC, n = 6), group II (BDL, n = 10), group III (SC+ 150 mg/kg Flu, n = 6) and group IV (BDL+ 150 mg/kg Flu, n = 10). BDL procedure was used to induce cholestasis. Briefly, the animals were anaesthetized with xylazine HCl (10 mg/kg) and ketamine HCl (50 mg/kg). Thus, after induction of anesthesia, the common bile duct of the rat was tied and cut off in the middle. This led to failure in bile secretion and led to cholestasis. Simultaneously, on a number of animals, all operations except the bile duct ligated were performed, and these rats were considered as SC rats. 24 h after surgery, the rats received 150 mg/kg Flu or normal saline via oral administration for 7 consecutive days. On 8th day, the blood was taken and centrifuged for 10 min (3000g); then, the plasma was collected and stored at −20 °C.

After collecting the blood, liver tissues were removed. The liver tissue allocated to three parts; homogenized in PBS (10 mmol/L, pH 7.4) for oxidative stress markers, kept in liquid nitrogen for mRNA expression, and stored in 10% formalin for histological examination.

### 2.3 Biochemical examination

Alkaline phosphatase (ALP), total bilirubin (TBIL), albumin (ALB), alanine aminotransferase (ALT), and aspartate aminotransferase (AST) were assayed by typical diagnostic kits (Bionik Diagnostic Co., I.R. Iran).

### 2.4 Determination of ferric reducing antioxidant power (FRAP)

This process is based on reducing ferric ions (Fe^3+^) and converting them to ferrous ions (Fe^2+^) at acidic pH and in the presence of TPTZ (Tripyridyls-s-triazine). The Fe-TPTZ complex had blue color. The resulting color can be measured spectrophotometrically at 593 nm [9].

### 2.5 Measurement of protein carbonyl (PCO) content

The plasma samples were mixed with 2, 4-dinitrophenylhydrazine reagent (10 mmol) in 2 M HCl for 60 min at 25 °C. 50% trichloroacetic acid (TCA) was added to the samples, then, after centrifugation it was washed with ethanol/ethyl acetate solution (1:1 ratio). Guanidine hydrochloride solution (6M) was added to the precipitate. Lastly, the optical density (OD) was read at 370 nm and the values of PCO was assayed utilizing the molar absorption coefficient (2.2 × 104 M^−1^ cm^−1^) [18].

### 2.6 Measurement of malondialdehyde (MDA)

Briefly, 500 λ of plasma and homogenate tissue were mixed with reagent including 15% w/v TCA, 0.375% w/v TBA, and 0.25 N HCl. The solution was kept for 30 min in a boiling water bath. Then, it was centrifuged for 5 min at 10,000 g and the OD was read at 535 nm [19].

### 2.7 Measurement of total thiols (tSH)

The levels of tissue and plasma tSH were assayed according to the interaction of DTNB with tSH groups. In brief, 25 μl of sample, 150 μl of Tris-EDTA, 10 μl of DTNB, and 790 μl of absolute methanol were mixed in the microtubes. They were gently kept at 25 °C for 15 min. The OD was read at 412 nm and the tSH value was assayed utilizing a molar absorption coefficient of 13600 M^−1^ cm^−1^ [20].

### 2.8 Measurement of nitric oxide (NO) metabolite

Nitric oxide metabolite was assayed based on the Griess reaction. Briefly, 200 μL of sample plus 100 μL of acetonitrile was centrifuged at 10000 g for 5 min. Then, 100 μL of the Griess reagent was mixed with 100 μL of supernatant and kept for 30 min at 37 °C. The OD was read at 540 nm [20].

### 2.9 Histopathological analysis

The histological examinations were done according to pervious study [21].

### 2.10 RNA extraction and real time PCR

Total RNA was separated from hepatic tissues utilizing RNX Plus (Sinaclon, Tehran, Iran) and cDNA was produced from total RNA by cDNA Synthesis kit (Sinaclon, Tehran, Iran) based on the manufacturer's procedure. RT-PCR was done by Rotor Gene 3000 instrument (Bio-Rad, USA), and data of TNF-α, α–SMA, IL-1, and TGF-β were determined in comparison to GAPDH using the 2^–ΔCt^ procedure.

### 2.11 Statistical analysis

Data were analyzed using one-way ANOVA test following Tukey's test. The results were showed as mean and standard error of mean (SEM). P ≤ 0.05 level was determined in all tests.

## 3 Results

### 3.1 Histopathological changes

**Figure 1:**
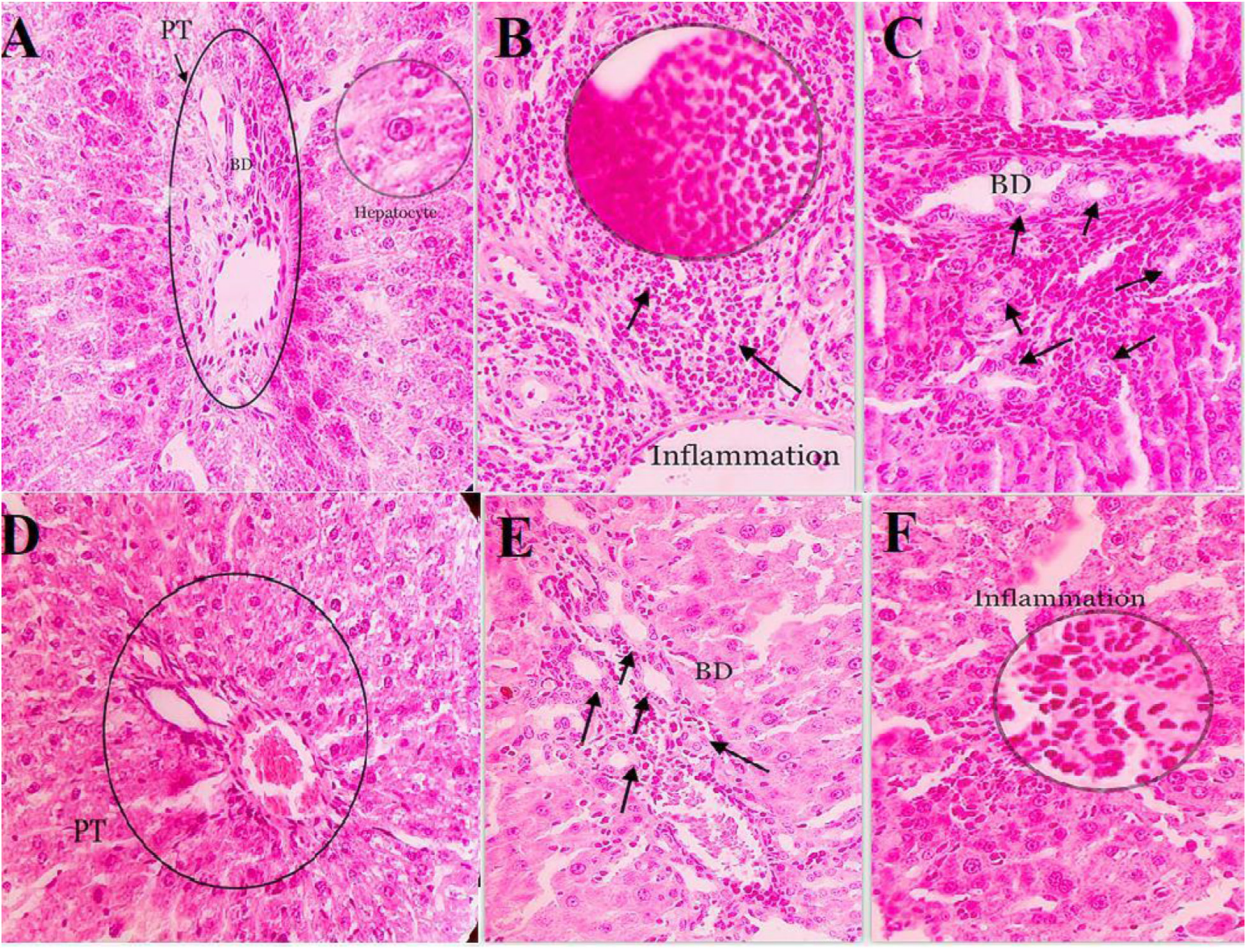
Histopathological results of liver tissue stained with hematoxylin and eosin (×10). (A) SC, (B, C) BDL, (D) SCF, (E, F) BDLF. SC, sham control; BDL, bile duct ligation; SCF, SC + fluvoxamine; BDLF, BDL + fluvoxamine; PT, portal tract; BD, bile duct.

Histologic sections indicated that SC and SCF groups had normal structures in liver tissue (Figure 1A and D). BDL rats showed severe biliary hyperplasia and portal inflammation, as well as mild hepatocellular damage (Figure 1B and C). The BDLF group showed improvement in tissue condition, but not complete relief as having mild to moderate biliary hyperplasia and portal inflammation, without hepatocellular damage (Figure 1E and F) (Table 1).

**Table 1:** The impact of Flu on histopathological examinations in BDL-induced cholestatic rats.

Group	Biliary hyperplasia	Portal inflammation	Hepatocellular damage	Vascular damage
SC	Zero	Zero	Zero	Zero
BDL	Severe	Severe	Mild	Zero
SCF	Zero	Zero	Zero	Zero
BDLF	Mild-to moderate	Mild-to moderate	Non	Zero

SC, sham control; BDL, bile duct ligation; SCF, SC + fluvoxamine; BDLF, BDL + fluvoxamine.

### 3.2 Biochemical parameters

Our results showed that the serum values of ALP, total bilirubin, AST, and ALT in the BDL rats were significantly increased in contrast to SC (P ≤ 0.05), while the value of ALB in the BDL group did not show a marked change in comparison to SC group. Injection of Flu in BDL rats slightly reduced ALT, total bilirubin, and ALP compared to the BDL alone group. Nevertheless, Flu was able to significantly increase ALP in comparison to BDL rats (P ≤ 0.05) (Table 2).

**Table 2:** The impact of Flu on plasma biochemical markers in BDL-induced cholestatic rats.

Groups	AST (U/L)	ALT (U/L)	ALP (U/L)	Total bilirubin (mg/dl)	ALB (mg/dl)
SC	148.00 ± 23.75	67.50 ± 11.22	805.33 ± 60.13	0.33 ± 0.11	3.03 ± 0.07
BDL	439.62 ± 72.23∗	171.00 ± 31.81∗	1847.25 ± 248.92∗	7.76 ± 0.89∗	2.92 ± 0.05
SCF	141.60 ± 21.30	58.20 ± 6.49	1119.20 ± 132.31	0.22 ± 0.02	3.06 ± 0.04
BDLF	517.14 ± 71.34	166.00 ± 22.41	1656.33 ± 161.26	7.27 ± 0.35	3.07 ± 0.02^#^

Values presented as mean ± SEM. Data were analyzed using one-way ANOVA test following Tukey's test. Statistically significant compared to SC group, ∗P ≤ 0.05; statistically significant in contrast to BDL group, ^#^P ≤ 0.05. BDL, bile duct ligation; SC, sham control; SCF, SC + fluvoxamine; BDLF, BDL + ffluvoxamine; ALP, alkaline phosphatase; TBIL, total bilirubin; AST, aspartate aminotransferase; ALT, alanine aminotransferase; ALB; albumin.

### 3.3 Oxidative stress parameters

As shown in Table 3, a significant increase in liver NO metabolite, liver MDA level, and plasma PCO were observed in the BDL alone rats in contrast to SC group (P ≤ 0.05), while injection of Flu in BDL rats markedly reduced the amount of these parameters against the BDL alone rats (P ≤ 0.05). Liver TSH levels in the BDL group was markedly reduced in contrast to SC rats (P ≤ 0.05), while injection of Flu in the BDL rats had not effect on it.

**Table 3:** The impact of Flu on the tissue oxidative stress parameters in BDL-induced cholestatic rats.

Groups	FRAPL	FRAPP	NOP	TSHL	TSHP
SC	44.52 ± 2.17	170.25 ± 35.83	40.85 ± 7.31	4.28 ± 0.44	17.81 ± 2.23
BDL	45.45 ± 4.17	827.43 ± 76.54∗	53.78 ± 11.31	2.78 ± 0.34∗	27.82 ± 1.69∗
SCF	31.11 ± 3.90	257.00 ± 55.53	32.08 ± 5.68	2.44 ± 0.14	14.73 ± 1.92
BDLF	33.71 ± 2.18^#^	768.06 ± 66.61	35.18 ± 8.60	2.59 ± 0.56	19.22 ± 0.97^#^

Values presented as mean ± SEM. Data were analyzed using one-way ANOVA test following Tukey's test. Statistically significant in contrast to control group, ∗P ≤ 0.05; statistically significant in contrast to BDL group, ^#^P ≤ 0.05. BDL, bile duct ligated rats; SC, sham control; SCF, SC + fluvoxamine; BDLF, BDL + fluvoxamine; FRAP, ferric reducing antioxidant power; TSH; total thiol, NO metabolite, nitric oxide metabolite, L; liver, P; plasma.

**Figure 2:**
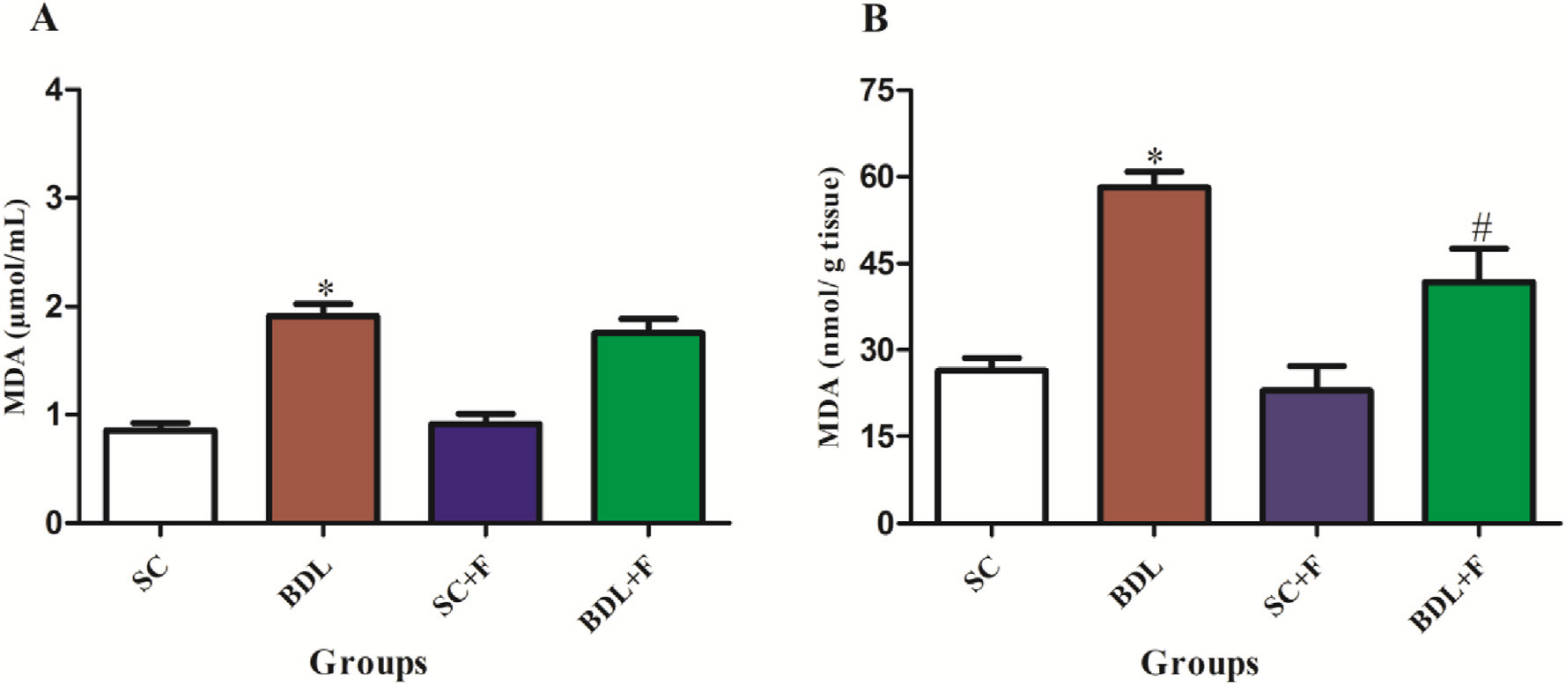
The impact of Flu on MDA content in the liver (A), and plasma (B) of BDL-induced cholestatic rats. Each content shows the mean ± SEM. Data were analyzed using one-way ANOVA test following Tukey's test. Statistically significant in contrast to control rats, ∗P ≤ 0.05, statistically significant in contrast to BDL rats, ^#^P ≤ 0.05. BDL, bile duct ligated rats; SC, sham control; SCF, SC + fluvoxamine; BDLF, BDL + fluvoxamine; MDA; malondialdehyde.

**Figure 3:**
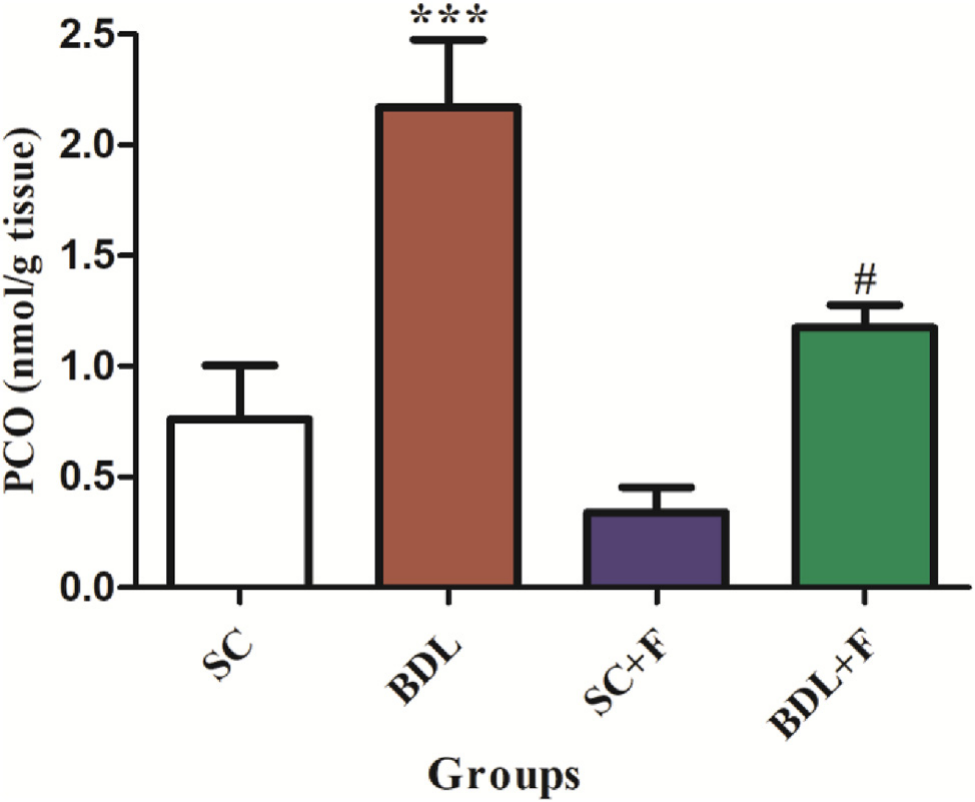
Effect of Flu on plasma PCO content in the BDL-induced cholestatic rats. Each content shows the mean ± SEM. Data were analyzed using one-way ANOVA test following Tukey's test. Statistically significant in contrast to control rats, ∗∗∗P ≤ 0.001, statistically significant in contrast to BDL rats, ^#^P ≤ 0.05. BDL, bile duct ligated rats; SC, sham control; SCF, SC + fluvoxamine; BDLF, BDL + fluvoxamine, PCO; protein carbonyl.

The liver and plasma MDA levels (Figure 2A and B), and tissue PCO (Figure 3), in BDL group showed a marked increase in contrast to SC animals, while injection of Flu in BDL rats markedly reduced the amount of these parameters (P ≤ 0.05).

### 3.4 RNA extraction and real time PCR

**Figure 4:**
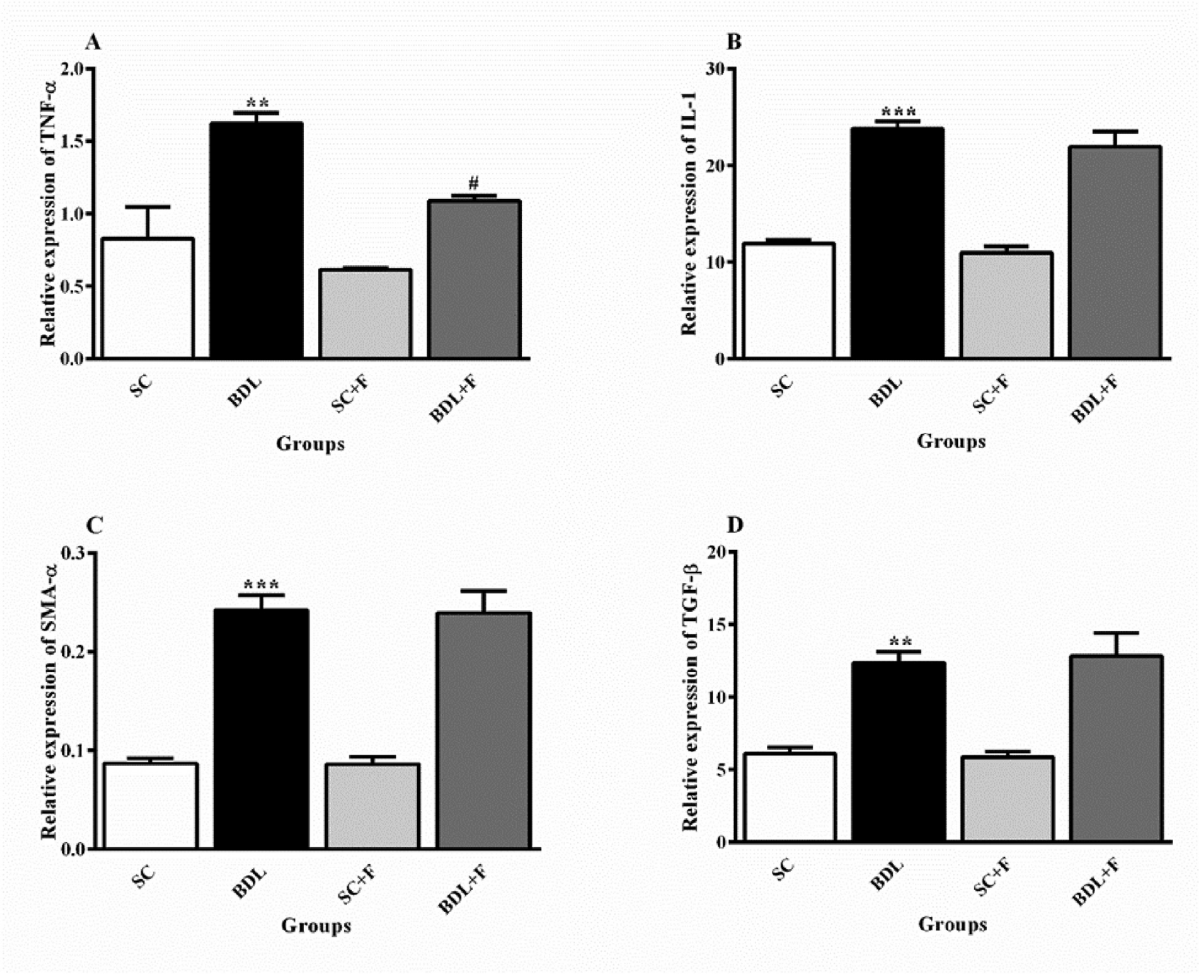
The impact of Flu on the mRNA levels of TNF-α, α-SMA, IL-1, and TGF-β in BDL-induced cholestatic animals. Each content shows the mean ± SEM. Data were analyzed using one-way ANOVA test following Tukey's test. Statistically significant in contrast to control rats, ∗∗P ≤ 0.01, ∗∗∗P ≤ 0.001; statistically significant in contrast to BDL animals, ^#^P ≤ 0.05. BDL, bile duct-ligated rats; SC, sham control; SCF, SC + fluvoxamine; BDLF, BDL + fluvoxamine.

The mRNA values of TNF-α, α –SMA, IL-1, and TGF-β markedly increased in the liver tissue of BDL rats in comparison to SC animals (P ≤ 0.05). Treatment with Flu significantly decreased the relative expression of TNF-α in contrast to the BDL alone rats (Figure 4 A-D).

## 4 Discussion

There is some evidence that indicate the vital role of ROSs in the pathogenesis of cholestasis and liver damage. The bile acid levels elevate in the BDL rats and stimulate lipid peroxidation, possibly by the induction of inflammatory cells and polymorphonuclear phagocytes [22]. The goal of this study was to assess the impacts of Flu on oxidant-antioxidant balance, liver function, and inflammatory cytokines in BDL cholestatic rats.

ALT, AST and ALP enzymes are used for diagnosis of hepatic damage, because they are released in the circulation after hepatocellular damage [23]. As estimated, BDL obviously elevated the liver function tests such as ALP, total bilirubin, ALT, and AST in the cholestatic rats. In accordance with these findings, Giménez et al. [24], and Kabirifar et al. [25] showed that serum values of ALP, AST, and ALT increased markedly in the BDL rats in contrast with the control rats. Also, Tahan et al. [26] and Ali et al. [27] showed that total bilirubin had a marked increment in the BDL rats in contrast with related control group. Our findings presented that Flu (150 mg/kg) had no marked impact on the ALP, ALT, and AST activities, and total bilirubin. In accordance with the present study, Tahan G and et al. [26] showed that there was no marked difference in ALB value in BDL group in contrast with control rats. However, treatment with Flu increased ALB level as compared to BDL rats. In harmony with biochemical markers, BDL induced morphological changes, such as biliary hyperplasia, portal inflammation, and hepatocellular damage, while treatment with Flu reversed partially these changes.

MDA is a secondary product during lipid peroxidation [28]. It is considered as a marker of tissue damage and reflect the degree of lipid peroxidation [29]. In agreement with our findings, liver MDA level elevates in BDL animal models [25, 30]. The values of tissue MDA in the Flu-treated group were markedly lower than BDL alone rats. Venlafaxine (VLF), an antidepressant, is a blocker of norepinephrine and serotonin. VLF exerted its protective effects against lipid peroxidation and oxidative stress via inhibiting noradrenaline and serotonin reuptake [31]. It seems that Flu inhibited the formation of MDA by stopping serotonin and noradrenaline reuptake.

Nitric oxide created in the liver by macrophages, hepatocytes endothelial cells, and kupffer cells in reaction to various stimuli. In accordance with the current study, Lim SW et al. [32], Esrefoglu et al. [33] showed that NO level had a marked increment in the BDL rats in contrast with the related control animals. Abdel-Wahab et al. [31] showed that VLF markedly declined NO levels by deactivating of reactive nitrogen species (RNS). We conclude that probably Flu inhibited the liver NO formation via neutralizing RNS.

Total thiol (tSH) is a vulnerable marker that has a major role in inactivating of ROSs [34]. In agreement with our findings, Kabirifar et al. [35], and Terzioglu et al. [36] indicated that the level of tSH was markedly reduced in the BDL rats in contrast with SC animals. However, Flu injection was not able to increase tSH. Glutathione is the main constituent of tSH groups, Flu may not be able to prevent glutathione consumption, or to increase its synthesis.

It seems that proteins are more susceptible to oxidative stress products, because of their role as enzymes in the cells [11]. The current study showed that the plasma PCO values were elevated in the BDL animals in contrast with the SC rats, whereas treatment with Flu (150 mg/kg) markedly decreased PCO values in comparison to BDL rats. Doustimotlagh et al. [37], and Sadeghi et al. [6] showed higher PCO levels in the BDL group in contrast to the SC rats. Zafir et al. [11] showed that antidepressants including Flu, VLF, and Imipramine, significantly reduced PCO levels in oxidative stress-induced rats which were in consistent with our study. These findings indicated that antidepressants generally preserve protein functions during the chronic stress.

Kupffer cells activation and inflammation play a major role in the fibrogenesis. The Kupffer cells produce pro-inflammatory cytokines such as IL-6, TNF-α, and IL-1, then, these cytokines effect on hepatic satellite cells [13]. Also, cytokines are involved in triggering and activating of hematopoietic stem cells, which produce collagen. Sheen et al. [38], and Tahan et al. [26] indicated that protein and mRNA values of TNF-α was markedly augmented in hepatic tissue of BDL rats which was in harmony with our results. The administration of Flu markedly ameliorated the level of TNF-α in contrast with the BDL animals. In harmony with this finding Ghareghani et al. [39] indicated that microglial cells stimulated by lipopolysaccharide increased the levels of TNF-α which were reduced by Flu. Based on anti-inflammatory activity of Flu, Flu may be reducing TNF-α level due to its anti-inflammatory properties.

## 5 Conclusion

The current study exhibited that Flu diminishes oxidative stress by inhibiting lipid and protein oxidation, as well as inhibiting of nitric oxide formation. Also, it reduced inflammation by decreasing TNF-α mRNA expression.

## Declarations

### Author contribution statement

Zahra Barmoudeh; Navid Omidifar; Zahra Moslemi: Conceived and designed the experiments; Performed the experiments.

Hossein Sadeghi: Performed the experiments; Contributed reagents, materials, analysis tools or data.

Izadpanah Gheitasi; Bahman Khalvati: Performed the experiments; Wrote the paper.

Mahdokht Azizi; Jafar Nikbakht: Analyzed and interpreted the data; Wrote the paper.

Amir Hossein Doustimotlagh: Conceived and designed the experiments; Performed the experiments; Contributed reagents, materials, analysis tools or data; Wrote the paper.

### Funding statement

Amir Hossein Doustimotlagh was supported by 10.13039/501100007119Yasuj University of Medical Sciences [960623].

### Data availability statement

Data will be made available on request.

### Declaration of interest's statement

The authors declare no competing interests.

### Additional information

No additional information is available for this paper.
